# Systematic and Collaborative Approach to Learning and Educational Content Development (SCALED) for Health Apps: An Experience-Informed Conceptual Framework

**DOI:** 10.2196/83442

**Published:** 2026-07-02

**Authors:** Qi Chwen Ong, Anita Pienkowska, Emina Obarcanin, Anne-Claire Stona, Andy W H Khong, Josip Car, Andy Hau Yan Ho

**Affiliations:** 1 Lee Kong Chian School of Medicine, Nanyang Technological University, Singapore, Singapore; 2 SingHealth Duke-NUS Global Health Institute, Duke-NUS Medical School, Singpapore, Singapore; 3 Centre for Regulatory Excellence, Duke-NUS Medical School, Singapore, Singapore; 4 School of Electrical and Electronic Engineering, Nanyang Technological University, Singapore, Singapore; 5 King’s Population Health Institute, King's College London, London, United Kingdom; 6 Psychology, School of Social Sciences, College of Humanities, Arts and Social Sciences, Nanyang Technological University, SHHK-04-03, 48 Nanyang Drive, Singapore, 639818, Singapore, 65 63168943

**Keywords:** patient education, mHealth, mobile health, health information, conceptual framework, health apps

## Abstract

Mobile health (mHealth) apps are widely used for noncommunicable disease prevention and self-management. However, their effectiveness and safety are undermined by substantial variation in content quality. Existing guiding frameworks primarily focus on user interface, functionality, and intervention delivery, with limited emphasis on content creation. In this viewpoint, we introduce Systematic and Collaborative Approach to Learning and Educational Content Development (SCALED), a conceptual framework designed to guide a systematic, collaborative, and evidence-based approach to mHealth educational content development, intended for app developers, researchers, health care providers, and the wider mHealth community. Developed and refined across 3 phases, the SCALED framework consists of 11 components organized into 3 sequential stages: planning and conceptualization, development of textual content, and finalization into delivery format. We discuss the rationale behind each component and illustrate its applicability through 2 mHealth use cases. The framework integrates real-world experience from the development of 3 mHealth apps, qualitative findings from 2 studies, and insights from key stakeholders. By offering a structured and replicable methodology for content development, SCALED addresses a critical gap in current mHealth frameworks and provides a practical guide to improve content veracity, with potential for adaptation across a range of medical conditions.

## Introduction

Mobile health (mHealth) apps have emerged as a promising tool for disease prevention and management, providing accessible, scalable, and personalized health care interventions. Defined by the World Health Organization as “medical and public health practice supported by mobile devices, such as mobile phones, patient monitoring devices, personal digital assistants, and other wireless devices” [1], mHealth apps are widely tested for patient education, remote monitoring, behavioral interventions, and personalized care [2].

As of 2024, it was estimated that more than 35,000 mHealth apps were available on both the Apple App Store and the Google Play Store [3,4]. Despite their ubiquity and growing adoption, the effect of these apps across various health outcomes remains inconsistent [2,5,6]. Evidence from systematic reviews indicates that most mHealth apps lacked clinical credibility [7], were developed without health care professionals’ (HCPs) involvement [8], and did not report theory or conceptual framework underpinning the development of educational materials [9]. Moreover, a scoping review found that 67 of 80 identified safety concerns with consumer-facing mHealth apps were related to the information quality, such as incorrect or incomplete information, variation in content quality, and inappropriate response to consumer needs [10].

These gaps could have a negative impact on their uptake, posing grave concerns for patient safety. Without adequate quality control measures and regulation for apps entering the market [11], health care providers and organizations may become more cautious and reserved about prescribing or recommending mHealth apps to patients [12]. From the user’s perspective, exposure to low-quality or misleading content may result in inappropriate lifestyle choices or self-management decisions, introducing potential clinical harms [13]. Given the dynamic and rapidly expanding mHealth landscape, it is imperative to establish and promote good practices in the design and development of educational content to ensure its effectiveness, safety, and trustworthiness.

## mHealth Frameworks and Instruments

A growing number of frameworks have been proposed for mHealth apps. These frameworks primarily focus on aspects related to technical and functional design [14,15], usability and user-centeredness [16,17], as well as risk and trustworthiness [13,18]. In parallel, many instruments and tools developed for mHealth apps are intended for quality assessment and rating purposes [19,20]. Notable examples include the Mobile App Rating Scale (MARS) [21], which is a multidimensional instrument for assessing app quality across domains such as engagement, functionality, aesthetics, and information quality, and the THESIS tool, which was developed to assess the risks and benefits of health apps across a broader set of dimensions [22]. Although most included criteria are related to the veracity of content, such considerations are often addressed in a generic sense only [19]. A systematic review of mHealth assessment frameworks synthesized evaluation criteria into 7 overarching categories, one of which pertains to information and content. Key elements in this category are credibility, accuracy, quality, and quantity of information [20]. This reflects the recognition of content quality, alongside technical design, usability, and data privacy, as a key factor in how mHealth apps are evaluated and ultimately used.

The importance of content veracity in mHealth has also been formally acknowledged. The CONSORT (Consolidated Standards of Reporting Trials)-eHealth checklist, which extends reporting standards for randomized controlled trials of web-based and mHealth interventions, explicitly recommends reporting on quality assurance measures taken to ensure accuracy and quality of information in eHealth interventions [23]. While tools for evaluating readability and quality of consumer-facing health information such as Flesch-Kincaid and DISCERN do exist [24,25], there remains a critical gap: the lack of standardized methodologies for developing mHealth educational materials that are evidence-based, clinically accurate, and theory-informed. In response, some articles have outlined multistage content development processes involving initial drafting, multiple rounds of edits, professional editing, and final reviews by clinical experts [26,27]. These offer useful starting points for shaping best practices in mHealth content development, but fall short of offering a structured, stepwise approach.

To address this gap, we propose a Systematic and Collaborative Approach to Learning and Educational Content Development (SCALED), a framework informed by real-world experience in developing 3 mHealth apps, qualitative insights from end-users, and iterative input from a multidisciplinary team. By integrating educational and behavioral change theories, clinical guidelines, and user-centered design principles, SCALED aims to provide a comprehensive guide for improving content credibility and accessibility. In this viewpoint, we describe the framework’s conceptual foundation, its application in 3 use cases, and its implications for mHealth research and practice.

## The SCALED Framework

### Conceptualization of the Framework

SCALED was conceptualized and developed in 3 phases. First, an initial framework informed by the development of a comprehensive mHealth education program was evaluated and improved in a co-design study with patients (GLOW) [28]. Second, application in 2 use cases (WellFeet and CADENCE D-PHA) to demonstrate its usability in practice and refine for improvement [29,30]. Third, finalization of the framework through discussion and consensus building with stakeholders involved in content creation. A glossary of key terms is provided in Textbox 1.

Textbox 1. **Glossary of Terms.**

**Theme:** broad, overarching topic that serves as a unifying thread for organizing curriculum content and learning experiences.

**Module:** self-contained units of educational content designed to address specific learning objectives within a theme.

**Bite-size:** small, manageable portions of content intended to facilitate focused learning and reduce cognitive load.

**Conversational agent (chatbot):** “Computer programs that use text, speech, and other input modalities to enable communication with users” [14].

**Action-oriented:** learning or intervention content designed to prompt users to perform specific behaviors, tasks, or decision-making steps that reinforce learning objectives.

### Research Context

The experience of developing a diabetes education app (GLOW, Nanyang Technological University Singapore) and findings from a qualitative co-design study with patients with lived experiences of diabetes guided the conceptualization of the initial mHealth content development framework [28]. GLOW was designed to provide users with verified health information on general knowledge related to Type 2 diabetes mellitus, including complications, foot health, medication, and blood glucose monitoring, and lifestyle behaviors including exercise, diet, and sleep. It consisted of 10 modules, comprising 63 units with learning cards, quizzes, mini-challenges, and rules-based action-oriented, frequently asked questions (FAQ) chatbot conversations. The content was guided by 172 knowledge-based, 54 skills-based, and 106 attitude-based learning objectives. Additionally, 232 audio recordings of the text, 711 static images, and 55 motion graphics were integrated to complement the textual content of the modules. Although patients were not directly involved, GLOW app content was co-created with lay professionals. These educational modules underwent review by HCPs, including endocrinologists, nurses, podiatrists, and dieticians, from a major tertiary hospital in Singapore. This process served as the basis for the initial structure of the SCALED framework.

We subsequently applied the initial framework to two use cases: (1) WellFeet, a multilingual diabetes foot care education app for individuals at risk of diabetic foot ulcers [29], and (2) CADENCE D-PHA (digital personal health assistant) app, a multicomponent mobile health app for improving medication adherence in adults with hyperlipidemia [30].

WellFeet content consists of 4 themes, 21 topics, and 54 bite-sized videos in English, Chinese, and Malay, accompanied by quiz questions and rules-based chatbot conversations. They were guided by 21 general, 54 knowledge-based, 40 skill-based, and 38 attitude-based learning objectives. Bite-sized animations with a voice-over and a synthetically generated artificial intelligence (AI) avatar-presenter were accompanied by quiz questions, polls, reflections, and rules-based action-oriented FAQ chatbot conversations framed by a personalized and adaptive learning experience. The content was reviewed by project co-investigators and HCPs from multiple major health care institutions in Singapore. The app was tested in a single-arm, pre-post mixed-methods feasibility study [29].

CADENCE D-PHA’s content (phase 1) comprises 5 themes, 13 topics, and 63 bite-sized videos accompanied by quiz questions and rules-based chatbot conversations. They were guided by 40 knowledge-based, 35 skill-based, and 20 attitude-based learning objectives. Bite-sized videos are animations with a voice-over and video recordings of HCPs and patients, accompanied by quiz questions, polls, free-text responses, and reflections. Similar to WellFeet, the education content is supported by rules-based, action-oriented FAQ chatbot conversations. Moreover, the content was reviewed by project co-investigators, collaborating HCPs from multiple tertiary and primary health care institutions in Singapore, as well as patient advocates. Phase 2 content is being developed and progressively rolled out with app updates. CADENCE D-PHA is currently being tested in an ongoing multicenter randomized controlled trial.

The framework was modified to meet the needs of each individual project, with components added or omitted as appropriate, considering factors such as target user groups, team composition, technological infrastructure, available funding, and project timeline. Applying the initial framework to these 2 use cases allowed us to assess the framework’s overall applicability and refine its components. Across the 3 use cases, several cross-cutting insights emerged that informed the refinement of the SCALED framework. First, the nature of deliverables differed across projects, ranging from text-based modules to multilingual videos and interactive chatbot features. Second, variations in available resources, including team composition and funding, shaped how specific components were operationalized, particularly in decisions related to outsourcing multimedia production. Third, differing technological requirements, such as the integration of conversational agents, multimedia elements, and adaptive features, introduced additional complexity in translating textual content into user-facing formats. Despite these differences, early-stage components such as team composition, theoretical grounding, and curriculum definition remained consistently critical, while challenges frequently arose at the interface between content creation and delivery. These cross-case insights highlight the importance of a structured yet adaptable framework and inform the final configuration of the SCALED components.

## SCALED Pillars

**Figure 1.**
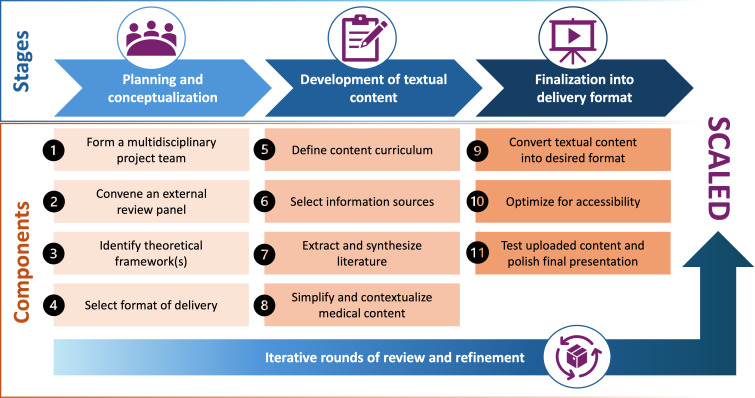
The Systematic and Collaborative Approach to Learning and Educational Content Development (SCALED) framework.

Drawing on real-world experience of developing 3 mHealth apps, 2 studies with end-users, and iterative discussions within the research team, the final SCALED framework (Figure 1) consists of eleven components embedded within 3 sequential stages. The framework moves from planning and conceptualization, to the development of textual content, and finally to its translation into delivery formats, reflecting the practical workflow observed across our 3 app development cases. The first stage establishes the conceptual, theoretical, and collaborative foundations of the intervention. The second focuses on generating, synthesizing, and tailoring the educational content, whereas the third addresses its conversion into accessible, polished, and implementation-ready materials.

The eleven components within SCALED were core recurring needs identified from our empirical experience, and they do not constitute an exhaustive taxonomy of all conceivable content-development activities. Although presented as a structured sequence, the components are not intended to function as rigid requirements to be applied identically in every context. Instead, they are core yet adaptable elements that may be modified, emphasized, or omitted depending on the intervention aims, target users, technological infrastructure, and available resources. Together, these components provide a structured pathway for developing mHealth educational content from initial conception to final deployment.

### Planning and Conceptualization

#### Form a Multidisciplinary Project Team

The creation of content in mHealth apps necessitates a multidisciplinary team to ensure clinical accuracy, behavioral and cultural relevance, and user-centered delivery. Team members from diverse professional backgrounds often bring differing expectations around project management, role responsibilities, content delivery, and even use distinct “language” (using different terminologies and methodologies), which can pose communication challenges [31]. However, the benefits of this diversity offset the potential difficulties, as varied backgrounds enable team members to complement one another’s knowledge and skills, ultimately enhancing the quality of the project.

HCPs contribute clinical expertise to ensure content alignment with clinical practice guidelines. Their involvement in mHealth development has been recommended to mitigate safety concerns [10]. Psychologists and behavioral scientists incorporate principles of behavior change to enhance the chances of healthy lifestyle modification. Learning designers provide input on content structure, educational framework, and delivery format to improve comprehension and accessibility. Although clearly defined roles should be established prior to initiating content development, in practice, many tasks require a flexible approach and a diverse set of competencies. For instance, content writers will often have responsibilities beyond script writing, such as adapting materials into animation-ready formats and coordinating the logistical aspects of video production. Depending on the scope and organizational capacity, specialized expertise may be acquired through external collaborators such as animators for creating visually engaging educational materials.

In all 3 cases, textual content was produced by a multidisciplinary team with capabilities to assess clinical information, conceptualize copywriting approaches, write relatable content, and perform project management duties. Given the multinational composition of the team, we made a deliberate effort to ensure our patient-centered message considered a balanced cultural power dynamic, avoiding dominance of any single cultural viewpoint. Multimedia components (eg, static graphics, animations, and short video recordings) were produced in-house by the project team in GLOW, drawing on existing expertise within the team and allowing close alignment with the pedagogical objectives. In contrast, for WellFeet and CADENCE D-PHA, a multimedia production unit was contracted to ensure professional quality, specialized technical skills, and efficient turnaround. The choice between in-house development and external contracting was guided by the availability of internal expertise, the complexity of the materials required, and project resources.

#### Convene an External Review Panel

It is important to engage stakeholders early in the process [32]. An external expert review panel is essential to ensure that consumer-facing health education materials are medically accurate, up-to-date, aligned with local (or regional) clinical practice and relevant to patients. In addition to the development phase, the review process should be repeated periodically as guidelines and patient needs evolve. The panel should include not just physicians, but also HCPs who are involved in the continuum of care, such as pharmacists, nurses, health psychologists, dietitians, and exercise physiologists. This allows every aspect of (chronic) disease management to be comprehensively reviewed from different perspectives.

It is equally important to actively involve a patient representative or advocate to ensure user-centered design [33]. Participatory action research (PAR) together with patient and public involvement (PPI) are increasingly recognized for their role in enhancing the quality and appropriateness of research [34]. In the context of mHealth, PAR and PPI could help identify end-user priorities, select pertinent health education topics, ensure linguistic and cultural appropriateness, and inform user-centered delivery formats. It is also recommended to involve individuals with lived experience of the condition to strengthen the impact of PAR and PPI [35].

In all 3 use cases, the content was reviewed by collaborators from partner health care institutions. For CADENCE D-PHA, we established a formal review panel independent of the content-writing team. It comprised primary care physicians, cardiologists, clinical pharmacists, health psychologists, dietitians, and patient advocates with lived experience of hyperlipidemia and cardiovascular disease (CVD). In the case of GLOW, we conducted a co-design study with a group of patients and caregivers with diverse socioeconomic and cultural backgrounds.

#### Identify Theoretical Framework

Establishing the theoretical underpinnings of an intervention is a fundamental step in the development of complex programs [32]. Despite this, most mHealth apps lacked a theoretical grounding in their development, including an educational component [8,9] and behavior changes. Identifying and selecting appropriate behavioral theory is essential for supporting the adoption of healthy behaviors, while educational frameworks guide a structured and outcomes-driven approach to learning. With a plethora of behavioral theories and health promotion strategies [36,37], selecting those most relevant to the intervention’s goals and the target population’s needs is critical to maximizing the effectiveness of mHealth interventions.

In our use cases, we selected the health belief model and the transtheoretical model because both were well-aligned with the educational and behavioral aims of the interventions [38,39]. The health belief model was useful for structuring content around risk perception, perceived benefits and barriers, and cues to action, whereas the Transtheoretical Model supported the stepwise tailoring of content to users’ readiness for behavior change. For instance, themes and topics in CADENCE D-PHA were systematically organized according to the core constructs of the health belief model. The content was designed to address the increased risk of developing CVD among patients with hyperlipidemia (perceived susceptibility), the impact of untreated hyperlipidemia and CVD on quality of life (perceived severity), misbeliefs and misconceptions about adverse effects of statins (perceived barriers), and the benefits of statin therapy in CVD prevention (perceived benefits).

However, theory selection within SCALED is not intended to be prescriptive. Rather, the framework encourages developers to identify and justify theoretical approaches that best fit the intervention goals, target behaviors, and characteristics of the intended user group. The theories used in our projects are therefore illustrative examples of how behavioral and educational frameworks can be applied within SCALED, rather than mandatory theoretical foundations of the framework.

#### Select Format of Delivery

The choice of instructional design and delivery format can influence user outcomes by engaging different learning styles and improving memory retention [40-42]. Compared to static images and text, animations have been shown to enhance procedural skills acquisition [43], with a positive impact on knowledge, attitudes, and cognitive engagement [44]. Conversational agents are also emerging as an innovative tool for patient education [14]. Nonetheless, the selection of delivery formats must balance the intervention’s goals with practical considerations, such as funding availability and access to skilled, experienced personnel.

Across our use cases, we progressively expanded the range of formats to support engagement and learning. Beginning with text, audio, and static images in GLOW, we subsequently incorporated animations in WellFeet. These animated videos were accompanied by an AI-generated avatar-presenter that delivered the scripts. In CADENCE D-PHA, we further included transcribed videos featuring HCPs and patients, as well as audio recordings for mindfulness and meditation practice. Conversational agents in the form of rules-based chatbots remained largely consistent across 3 mHealth apps, with adaptations in their placement within the overall learning sequence to suit the specific context of each intervention. The chatbots were designed to engage users in action-oriented conversations with branching logics, enabling some degrees of personalization of content based on users’ responses.

### Development of Textual Content

#### Define Content Curriculum

Learning objectives serve as a compass for educational content developers [45]. As content creation is iterative and often collaborative, a clearly defined curriculum acts as a blueprint to ensure logical flow, alignment with intervention goals, and consistency across contributors. Topics identified through clinical expertise and literature review can be organized into thematic groups to shape the learning outcomes that reflect the overarching goals of the intervention. Learning objectives and topics can be evaluated for completeness and quality using tools such as the DISCERN instrument [25]. Feedback from an external review panel further allows content writers to prioritize practical, relevant content and remove concepts that are overly theoretical or clinically complex.

In all 3 cases, we used Bloom’s taxonomy to structure learning progression and scaffold educational content by organizing it across two key dimensions: cognitive complexity and behavioral focus [46]. This involved guiding users from foundational knowledge acquisition (eg, recalling the symptoms of diabetes) to higher-order thinking skills such as creating and evaluating action-oriented goals. Complementing this, we adapted the competency-based education model to further categorize learning outcomes into knowledge, skills, and attitudes, ensuring a competency-driven approach with a strong emphasis on building efficacy in self-care skills and behaviors [47,48]. This scaffolding informed the development of learning objectives and encouraged deeper reflection on the complexity of targeted behaviors and their component tasks. It also supported more realistic decision-making about the cognitive and behavioral demands placed on users, ensuring an appropriate balance between expectation, ambition, and feasibility.

#### Select Information Sources

An evidence-based approach to content creation is critical to ensuring users receive health information that is up-to-date and clinically accurate. In line with principles of evidence-based medicine [49], information sources should be systematically identified and appraised. Primary sources should include an informed integration of guidelines from international or regional professional bodies, health agencies, and local clinical practice protocols. This is because although widely accepted international guidelines are useful, they might not serve the needs of certain populations or align with local standards. For instance, the recommendations for management of hyperlipidemia are known to differ between guidelines from Singapore’s Agency for Care Effectiveness [48], the European Society of Cardiology [50], and the American Heart Association/American College of Cardiology [51]. Research publications, such as randomized controlled trials and systematic reviews, should be referenced with caution as individual studies may yield conflicting conclusions, vary in methodological robustness, and lack clear recommendations for real-world patient care [52,53]. Local clinical practice protocol that integrates evidence from high-quality studies with expert consensus could render them more appropriate to inform patient education. If additional information on resources such as community care support and services is needed [54], websites of health authorities, organizations, and institutions such as the World Health Organization, the UK’s National Health Service, and the Centers for Disease Control and Prevention could be invaluable [48,55,56]. Once compiled, these guidelines serve as a repository to inform the entire content writing process. For each project, we systematically documented all sources used in content development, both for internal transparency and to provide users with credible references for further reading. This practice also aligns with the requirements of Google Play, which mandates the inclusion of references prior to publishing mHealth apps [57].

#### Extract and Synthesize Medical Literature

After creating a repository of information sources, the next step involves extracting information for the proposed topics and distilling key findings. Synthesis goes beyond merely compiling information. Given the variability in clinical guidelines and emerging evidence, it is essential to critically appraise recommendations and study findings, reconciling differences when necessary. It also involves integrating insights across multiple sources to present a balanced, contextually relevant perspective. This ensures that content remains aligned with best practices while being adaptable to specific context.

To minimize discrepancies among content writers and ensure consistency of outputs, we developed a structured template for content extraction and synthesis. This template was applied uniformly across all topics, detailing the topic description, learning outcomes, sources of evidence, raw information extracted, lay-adapted content, and the content review process. This standardizes the internal process of how and from where clinical recommendations, evidence summaries, and practical applications are documented, ensuring uniformity in tone, depth, and language. Apart from strengthening the overall coherence, the template also streamlines the review process, enabling the review panel to identify any misinterpretation of clinical recommendations by comparing the lay-adapted content with the original references and raw information extracted.

#### Simplify and Contextualize Medical Content

Translating complex medical information into accessible and relatable language is essential for effective health promotion [58]. Achieving this requires both simplification and contextualization. Simplification improves readability, which is a key determinant of health information accessibility, especially for people with low health literacy [59]. However, research indicates that the content of many mHealth apps was written at reading-grade levels that exceed recommended standards [60,61].

Adapting medical content into lay language involves not only enhancing readability by replacing medical jargon with simpler terms or simplifying sentence structure [59,62], but also adopting strategies to improve understandability and actionability [63], minimize linguistic stigma [64], and improve cultural sensitivity through enhancement of appropriateness and representativeness [65,66]. For example, recommendations such as the Mediterranean diet or olive oil use may hold little relevance for patients in Asia, as they do not align with local dietary norms.

For all use cases, we developed a copywriting guide with suggestions for simplified, actionable, and empathetic language. For WellFeet and CADENCE D-PHA, we adapted content into a conversational tone to suit the video scripts. We also used copywriting techniques, such as analogies and metaphors, to clarify complex ideas. When medical terms were necessary, they were supported by a built-in glossary, allowing users to click on highlighted terms for plain-language definitions. In CADENCE D-PHA, we also provided a short video introducing and explaining medical terminologies that were commonly used, such as low-density lipoprotein cholesterol, unsaturated fats, statins, and cardiovascular disease.

### Finalization Into Delivery Format

#### Convert Textual Content into Desired Format

Design principles differ across instructional formats, with videos requiring their own specific approach [67]. Adapting text-based content for video involves more than converting it into a script. It also calls for additional guidance and materials, especially since animators usually do not have a medical background and may need support in visualizing some of the medical concepts accurately. An iterative process of providing input, reviewing storyboards, and refining video drafts is essential to ensure clarity, accuracy, and effective communication.

For both WellFeet and CADENCE D-PHA, we collaborated closely with the appointed multimedia units, not only to guide the creative process in choosing color schemes, typography, and voice-overs, but also to ensure the visual content accurately reflected the intended message. Providing carefully selected reference images was essential for ensuring accurate depiction of anatomical structures and biological processes, such as cholesterol deposition and plaque formation in arteries. All videos underwent additional rounds of review by the content development team to verify accuracy, ensure consistency, and maintain alignment with the educational goals before being finalized and uploaded. After receiving the storyboards from animators, we optimized the characters and images. The attire, skin tone, and facial expression of animated characters were revised to better align with local context and reflect the diversity of the local population. By incorporating these considerations, mHealth apps could deliver inclusive, equitable, and accessible content to a broad range of users.

#### Optimize for Accessibility

Optimizing mHealth app content for better accessibility ensures that users with varying levels of digital literacy can effectively engage with the materials. Beyond app functionalities such as implementing adjustable font sizes or using a larger font as the default [68], content designers should consider including alternative text descriptions for images, high-contrast color schemes, and scalable fonts to accommodate users with visual impairments. For quizzes with text response in CADENCE D-PHA, we integrated a dictation tool to enable voice input, reducing the reliance on typing for individuals with fine motor impairments, visual disabilities, or cognitive difficulties such as dyslexia. For videos, we provided subtitles and transcripts to support individuals with hearing difficulties or impairments. This could also cater to users of a reading-based learning style. Voice-over texts were carefully refined for appropriate speed, tone, and accent suited for the target population.

#### Test Uploaded Content and Polish Final Presentation

Pilot testing of the final content is essential to optimize content delivery and ensure fitting with user interface before rolling out the mHealth app. The layout of content can impact visual perception and usability, often requiring refinement due to misalignment between content design and software development, which are frequently managed by separate teams. Factors such as user settings (eg, dark vs light themes and adjustable font sizes) can further influence how content is displayed and received. At this stage, established app evaluation and usability frameworks may also be used as complementary quality-assurance tools to assess broader aspects of app usability and overall quality beyond content development alone. In all 3 use cases, we observed such misalignments and addressed them during the refinement phase. For seamless presentation and usability, pilot testing of the app and its content should be conducted with end users and other stakeholders. Usability testing should gather feedback on navigation, content comprehension, visual compatibility, and overall user satisfaction. At this stage, established app evaluation and usability frameworks may also be used as complementary quality-assurance tools to assess broader aspects of app usability and overall quality beyond content development alone. Importantly, this feedback loop must be maintained continuously throughout the content development process to identify and address issues arising at every stage, ensuring that the final product meets user needs and delivers a polished, engaging experience.

## Discussion

SCALED is a conceptual framework that integrates insights from real-world experience of developing mHealth apps, findings from 2 studies with end-users, and adaptations based on stakeholders’ input. With eleven components grouped into 3 sequential stages, the framework offers a practical guide for mHealth content development. Unlike existing frameworks that primarily focus on app usability and intervention design, SCALED provides a structured approach to producing mHealth content that is evidence-based, clinically accurate, theoretically grounded, and user-centered. This is particularly important given that many mHealth apps currently on the market lack health care professional involvement and fail to integrate behavioral science principles, potentially limiting their effectiveness in promoting sustained behavior change.

Concerns about the content quality and safety of mHealth apps are not new. Previous research has attempted to overcome this challenge by proposing various tools and frameworks to establish standards for evaluating the content, effectiveness, and usability of mHealth apps [69]. A range of frameworks has also emerged to guide specific aspects of mHealth, such as smartphone-delivered conversational agents [14], behavior intervention technology design [70], user-centered technology design [17,33], and data security and privacy [71,72]. In parallel, broader frameworks such as the Medical Research Council framework and the Behavioral Intervention Technology model provide high-level guidance on the development and evaluation of complex interventions and on aligning clinical aims with behavior change strategies and technological features [32,70]. SCALED is intended to complement, rather than supersede, these frameworks by addressing a more specific but underdeveloped aspect of mHealth intervention design: the systematic development of educational content. This directly tackles the root cause of variability in content quality.

SCALED may have broad implications for stakeholders in mHealth apps. For developers, it provides a structured methodology to create clinically accurate, theoretically grounded, and user-centered educational content, which could help build user trust and support regulatory compliance [73]. This could be highly relevant in commercial settings, where a multidisciplinary team of HCPs might not be readily available within the organizations. In such cases, SCALED’s recommendation to engage an external review panel could ensure content credibility and alignment with clinical standards. Users could benefit from more reliable, accessible, and evidence-based health information, enhancing their health literacy and informed decision-making [74]. Health care providers could use SCALED as a reference to appraise the rigor of the mHealth content development process and to assess their appropriateness before recommending them to patients. Additionally, for health systems actors and policymakers, the framework could serve as a foundation for establishing quality standards and guiding endorsement of digital health tools. By promoting a more consistent and transparent approach to content development, SCALED may ultimately support improvements in the effectiveness, credibility, and uptake of mHealth apps across different levels of the digital health ecosystem.

A key strength of the SCALED framework is its empirical foundation, having been refined based on real-world lessons from 3 mHealth apps, making it more practical and applicable than purely theoretical frameworks. Additionally, SCALED benefited from iterative feedback loops involving key stakeholders, including patients, HCPs, and content designers. Furthermore, its high-level structure provides a flexible foundation, allowing room for modification and customization of the more intricate steps in developing content across different formats and disease contexts.

A few limitations should be acknowledged. One major limitation is its untested generalizability, as the framework is based on a limited number of education-focused mHealth apps targeting chronic disease management, which may limit its direct applicability to other contexts. For instance, interventions in acute care settings, mental health, or those incorporating highly adaptive or real-time decision-making may require additional considerations beyond those captured in SCALED. Similarly, while the framework is designed to be flexible, its implementation in low-resource settings or by commercial developers without access to multidisciplinary academic teams may present practical challenges. SCALED is currently most directly applicable to interventions with a strong educational component, particularly those involving structured or rule-based content and human-reviewed materials. Its applicability to systems that rely heavily on generative AI may be limited, thus requiring further adaptation to address issues such as dynamic content generation, validation, and real-time personalization.

Another limitation of the framework is that it does not take into account regulatory checkpoints or alignments, as digital health policies vary globally. While SCALED does not address formal requirements set by regulatory bodies such as the United States Food and Drug Administration and CE (Conformité Européene) marking processes in Europe, it may serve as a complementary, preregulatory quality assurance approach by supporting the development of evidence-based and transparent educational content. It is worth noting that the framework was designed to serve as an initial step in response to the urgent need for improved quality of content in mHealth apps. Future iterations could be strengthened by having stronger engagement of target user groups and incorporating findings from systematic reviews, qualitative research, and structured stakeholder consensus methods, such as a Delphi study, to enhance framework comprehensiveness and applicability.

## Conclusions

The SCALED framework offers a systematic, collaborative, and evidence-based approach to mHealth content development, addressing a critical gap in existing mHealth offerings. By ensuring alignment with clinical guidelines, theoretical frameworks, behavioral science principles, and user-centered design, SCALED demonstrates a practical and stepwise approach to improving the quality of educational content and safety of mHealth apps. Future work may focus on assessing its usability across diverse development settings, evaluating its influence on content quality and user learning, and exploring its potential for adaptation in different health contexts and digital ecosystems.

## Abbreviations

AI: artificial intelligence
CONSORT: Consolidated Standards of Reporting Trials
CVD: cardiovascular disease
FAQ: frequently asked questions
HCP: health care professional
MARS: Mobile App Rating Scale
mHealth: mobile health
PAR: participatory action research
PPI: patient and public involvement
SCALED: Systematic and Collaborative Approach to Learning and Educational Content Development
